# Regulation of c-Fos Gene Expression by NF-κB: A p65 Homodimer Binding Site in Mouse Embryonic Fibroblasts but Not Human HEK293 Cells

**DOI:** 10.1371/journal.pone.0084062

**Published:** 2013-12-30

**Authors:** Yu-Cheng Tu, Duen-Yi Huang, Shine-Gwo Shiah, Jang-Shiun Wang, Wan-Wan Lin

**Affiliations:** 1 Department of Pharmacology, College of Medicine, National Taiwan University, Taipei, Taiwan; 2 National Institute of Cancer Research, National Health Research Institutes, Zhunan, Miaoli County, Taiwan; 3 Graduate Institute of Medical Sciences, Taipei Medical University, Taipei, Taiwan; South Texas Veterans Health Care System and University of Texas Health Science Center at San Antonio, United States of America

## Abstract

The immediate early gene c-Fos is reported to be regulated by Elk-1 and cAMP response element-binding protein (CREB), but whether nuclear factor (NF)-κB is also required for controlling c-Fos expression is unclear. In this study, we determined how NF-κB’s coordination with Elk/serum response factor (SRF) regulates *c-fos* transcription. We report that PMA strongly induced c-Fos expression, but tumor necrosis factor (TNF)-α did not. In mouse embryonic fibroblasts, the PMA induction of c-Fos was suppressed by a deficiency in IKKα, IKKβ, IKKγ, or p65. By contrast, in human embryonic kidney 293 cells, PMA induced c-Fos independently of p65. In accordance with these results, we identified an NF-κB binding site in the mouse but not human *c*-*fos* promoter. Under PMA stimulation, IKKα/β mediated p65 phosphorylation and the binding of the p65 homodimer to the NF-κB site in the mouse *c-fos* promoter. Furthermore, our studies demonstrated independent but coordinated functions of the IKKα/β-p65 and extracellular signal-regulated kinase (ERK)-Elk-1 pathways in the PMA induction of c-Fos. Collectively, these results reveal the distinct requirement of NF-κB for mouse and human *c-fos* regulation. Binding of the p65 homodimer to the κB site was indispensable for mouse *c-fos* expression, whereas the κB binding site was not present in the human *c-fos* promoter. Because of an inability to evoke sufficient ERK activation and Elk-1 phosphorylation, TNF-α induces c-Fos more weakly than PMA does in both mouse and human cells.

## Introduction

The immediate early gene c-Fos is a protooncogene that can dimerize with c-Jun to form an activator protein (AP)-1 transcription complex [1]. c-Fos plays crucial roles in the molecular mechanisms underlying a variety of cellular processes including proliferation, differentiation, transformation, and apoptosis [2]. Conversely, deregulation of c-Fos may be linked to diverse pathological conditions including immunological, skeletal, and neurological defects, as well as to oncogenic transformation and tumor progression. c-Fos determines the functions of osteoblasts and osteoclasts, and regulates cytokine release in inflammatory diseases [3]. Overexpression of c-Fos in animals results in chondroblast and osteoblast transformation, which contribute to osteosarcoma formation. In contrast to the widespread view on c-Fos’s oncogenic function, c-Fos also serves critical apoptotic and tumor-suppressive functions by upregulating Bim [4], [5]. Moreover, mice lacking c-Fos expression have been reported to exhibit abnormal development and function in certain tissues [6].

c-Fos expression is primarily controlled by mitogens acting through signal-induced phosphorylation of transcription factors, ternary complex factor (TCF)/Elk-1, serum response factor (SRF), and cAMP response element-binding protein (CREB) [7]. Accumulating evidence indicates that the phosphorylation of Elk-1′s transactivation domain critically regulates the ternary complex formation of Elk-1 with SRF, which occurs through the binding to the serum response element (SRE) within the *c-fos* promoter, and, thus, phosphorylation serves as a prerequisite for the transactivation function of Elk-1. The transactivation domain of Elk-1 is phosphorylated in most cases by extracellular signal-regulated kinase (ERK) [1], [8], [9], although the phosphorylation may also be mediated by p38 and c-Jun N-terminal kinase (JNK), depending on the stimuli encountered [10]. SRF is also phosphorylated, and its phosphorylation by ERK, CaMKK, or PI3K was reported to increase *c-fos* expression [11]–[13]. Furthermore, SRF-mediated *c-fos* expression has been reported to be positively regulated by the transcriptional co-activator MKL1 and the RhoA-dependent pathway [14]. In addition to the SRE, the CREB-response element (CRE) is required for maximal *c-fos* promoter activation. The phosphorylation of CREB at Ser133 by numerous kinases including RSK, MSK, protein kinase C (PKC), and Akt also plays a crucial role in *c-fos* transcription [15]. Among these kinases, MSK has the highest affinity for CREB and acts as the primary regulator of CREB activity. Moreover, in addition to SRF-dependent recruitment of Elk-1 to the SRE, Elk-1-mediated recruitment of ERK and MSK to SRE promoter complexes has been reported [16].

Nuclear factor (NF)-κB is a key transcription factor that is tightly regulated by diverse signaling pathways and controls the expression of numerous genes [17],[18]. Although NF-κB-dependent regulatory mechanisms acting through Elk-1 and SRF have been well characterized, functional studies linking NF-κB activation to *c-fos* expression are limited. Elk-1 was previously identified as a downstream target gene of NF-κB, and this function of NF-κB consequently contributes to c-Fos expression induced by serum and other stimuli [19]. Another study using epidermal growth factor (EGF)-stimulated fibroblasts as a model demonstrated that IKKα and p65/RelA contribute substantially to EGF-induced *c-fos* expression independently of the classical IκBα degradation pathway [20]. However, whether the classical NF-κB pathway also similarly upregulates *c-fos* expression remains unknown. Moreover, whether the NF-κB signal alone is sufficient for inducing c-Fos or whether NF-κB signaling must be functionally coordinated with the ERK pathway to induce c-Fos has not been investigated.

In this study, we addressed the role of the IKK-dependent NF-κB pathway and its crosstalk with ERK and downstream transcription factors (Elk-1, SRF, and CREB) in the control of *c-fos* expression. We compared and analyzed the effects and molecular mechanisms of action of 2 stimuli, phorbol 12-myristate 13-acetate (PMA) and tumor necrosis factor (TNF)-α, which were shown to activate NF-κB and ERK signaling to distinct degrees in diverse cell types. We report that a κB site is present in the mouse, but not human, *c-fos* promoter, and also that p65 homodimer binding to the *c-fos* promoter and Elk-1 activation must be coordinated to regulate mouse *c-fos* transcription. An inability of TNF-α to evoke sufficient ERK activation and the consequent low-level Elk-1 phosphorylation make TNF-α a weak c-Fos inducer compared with PMA.

## Materials and Methods

### Cell Cultures

Human embryonic kidney (HEK) 293 cells were obtained from American Type Culture Collection (Manassas, VA, USA). Wild-type (WT), IKKα^−/−^, IKKβ^−/−^, IKKγ^−/−^, and p65^−/−^ mouse embryonic fibroblasts (MEFs) were a kind gift from Dr. Michael Karin (University of San Diego, San Diego, CA, USA) [21]–[24]. HEK293 and MEFs were cultured in complete Dulbecco’s modified Eagle medium (DMEM) containing 10% (v/v) heat-inactivated fetal bovine serum (FBS), 100 U/mL of penicillin G, and 100 µg/mL of streptomycin. Cells were incubated at 37°C in a humidified incubator with 5% CO_2_ mixed in the air.

### Reagents

DMEM, FBS, trypsin-EDTA, penicillin G, and streptomycin were purchased from Invitrogen (Rockville, MD, USA). Protease inhibitor cocktails were purchased from Sigma Aldrich (St. Louis, MO, USA). PMA, MG132, U0126, SB203580, Ro318220, Ro320432, and LY333531 were obtained from Calbiochem (La Jolla, CA, USA). Mouse and human TNF-α were obtained from PeproTech (Rocky Hill, NJ, USA). SP600125 and H-89 were obtained from Tocris Cookson (Ellisville, MO, USA). BI-D1870 was purchased from Enzo Life Sciences (Plymouth Meeting, PA, USA). Antibodies directed against IKKγ, ERK1, ERK2, JNK1, p38, c-Fos, p50, p65, RelB, and c-Rel, and horseradish peroxidase (HRP)-conjugated antirabbit and antimouse antibodies were purchased from Santa Cruz Biotechnology (Santa Cruz, CA, USA). Antibodies against p100-p52, IKKα, IKKβ, Elk-1, SRF, CREB, and phosphorylated forms of SRF (Ser103), IKKα (Ser176)/ IKKβ (Ser177), p65 (Ser536), IκBα (Ser32), Elk-1 (Ser383), CREB (Ser133), JNK (Thr183/Tyr185), ERK (Thr202/Tyr204), and p38 (Thr180/Tyr182) were obtained from Cell Signaling Technology (Beverly, MA, USA). Antibodies against IKKγ (for kinase assays) and β-actin were purchased from BD Bioscience (Franklin Lakes, NJ, USA). Plasmids of GST-IκBα (1–67) and HA-p65 were provided by Dr. Michael Karin and Dr. Hiroaki Sakurai, respectively. [γ-^32^P] ATP was purchased from NEN (Boston, MA, USA).

### Immunoblotting Analysis

After the cells were treated with reagents, the medium was aspirated and the cells were rinsed once with ice-cold phosphate-buffered saline (PBS) and then harvested and lysed directly using a 1× sodium dodecylsulfate (SDS) gel-loading buffer (50 mM Tris-HCl at pH 6.8, 2% 2-mercaptoethanol, 0.1% bromophenol blue, and 10% glycerol). Samples were heated at 95°C for 5 min, and equal amounts of whole-cell extracts were subjected to SDS-polyacrylamide gel electrophoresis (PAGE; 8%–15%) and then transferred to Immunobilon-P (Millipore). Membranes were blocked using TBST (50 mM Tris-HCl at pH 7.5, 150 mM NaCl, and 0.1% Tween 20) containing 5% nonfat milk for 1 h at room temperature to minimize nonspecific antibody binding. After hybridization with the primary antibodies, the membranes were washed 3 times with TBST and incubated with HRP-conjugated secondary antibodies for 1 h. After washing 3 times with TBST, protein bands were detected by using an enhanced chemiluminescence (ECL) detection reagent (PerkinElmer or Millipore). β-Actin was used as an internal control.

### In vitro IKK Assay

Cell lysates were precleaned at 4°C for 1 h and then mixed with 0.5 µg of an anti-IKKγ antibody overnight. Subsequently, 10 µL of protein A-agarose beads were added and the samples were placed on a rocker for 1 h at 4°C. The immunoprecipitates were washed 5 times with a cold lysis buffer and then twice with a kinase reaction buffer (20 mM HEPES at pH 7.5 and 10 mM MgCl_2_). The beads were then incubated at 30°C in 40 µL of a kinase reaction buffer supplemented with 10 µCi of [γ-^32^P] ATP, 2 mM NaF, protease inhibitor cocktails, and 2 µg of GST-IκBα. The reaction products were separated using SDS-PAGE and then examined using autoradiography. GST-IκBα (1–67) was prepared by transforming *Escherichia coli* strain JM109 with a plasmid encoding the GST-fusion protein. The GST-fusion protein was induced and purified according to a commercial kit protocol (Bulk GST Purification module, GE Healthcare UK Limited, Amersham Place, Little Chalfont, Buckinghamshire, UK).

### Preparation of Nuclear Extracts and Electrophoretic Mobility Shift Assay (EMSA)

Nuclear extracts were prepared from MEFs as previously described [25]. Each gel-shift reaction mixture contained 0.025 pmol of a radiolabeled probe, 1 µg of poly (dI-dC), and 5 µg of nuclear proteins in a buffer of 50 mM NaCl, 30 mM KCl, 10 mM Tris-HCl at pH 7.5, 1 mM EDTA, 1 mM DTT, and 10% glycerol. Competing DNA oligonucleotides and antibodies against p65, p50, p52, RelB, and c-Rel (Santa Cruz Biotechnology) were added to the nuclear extract and incubated for 30 min on ice prior to the DNA binding reaction. After the DNA binding reaction was allowed to proceed at room temperature for 25 min, the samples were electrophoresed through non-denaturing 4% polyacrylamide gels for 1.5 h at 70 V. The gels were then dried and used for autoradiography. Competition experiments were performed by adding 1 pmol of an unlabeled probe. The oligonucleotides used in this study included the NF-κB-binding site in mouse *c-fos* (−226 to −196 bp): 5′-GGA AGG TCT AGG AGA CCC CCT AAG ATC CCA-3′; the NF-κB consensus binding sequence: 5′-AGT TGA GGG GAC TTT CCC AGG C-3′, the AP-1 consensus binding sequence: 5′-CGC TTG ATG ACT CAG CCG GAA-3′, and the NF-Y consensus binding sequence: 5′-CTG ATT GGY YRR-3′.

### Quantitative Polymerase Chain Reaction (qPCR)

The cells were harvested using RNA-Bee isolation reagents (Roche, Mannham, Germany) and total RNA was extracted by following the manufacturer’s protocol. We synthesized cDNAs by using MMLV reverse transcriptase purchased from Promega (Madison, WI, USA). To quantify mRNA levels, we performed real-time PCR by using an ABI Prism 7900 (Applied Biosystems, Foster City, CA, USA) and then detected the fluorescence of samples in 96-well plates by using FastStart SYBR Green Master (Roche, Mannham, Germany). Each 25-µL PCR reaction contained the cDNA, the Master Mix, and the following primers: the *c-fos* forward primer 5′-CGA AGG GAA CGG AAT AAG ATG-3′ and reverse primer 5′-GCT GCC AAA ATA AAC TCC AG-3′; and the β*-actin* forward primer 5′-CGG GGA CCT GAC TGA CTA CC-3′ and reverse primer 5′AGG AAG GCT GGA AGA GTG C-3′.

### Immunofluorescence Microscopy

The cells were seeded in 96-well plates and grown until they were 70%–80% confluent. After stimulating with indicated reagents, the medium was aspirated and the cells were fixed using 4% paraformaldehyde in PBS (pH 7.4) for 15 min at room temperature. The samples were washed 3 times with PBS and then incubated for 10 min in PBS containing 0.25% Triton X-100. The cells were then washed in PBS 3 times and incubated for 1 h in PBST containing 1% bovine serum albumin (BSA) to block nonspecific binding of antibodies. The cells were incubated overnight at 4°C with the anti-p65 primary antibody, washed 3 times using PBS (5 min for each wash), and then incubated with FITC-conjugated secondary antibodies diluted in a buffer containing BSA (1%) and DAPI (0.1 µg/mL). After being incubated for 1 h at room temperature in the dark, the cells were washed 3 times with PBST and then subjected to immunofluorescence microscopy to determine the cellular localization of p65.

### *c-fos* Promoter and the NF-κB Activity Assay

To construct the *c-fos* promoter, we isolated genomic DNA from C57BL/6 mice and amplified the promoter region from +30 to −1070 by performing PCR using KOD hot-start DNA polymerase (Merck KGaA, Darmstadt, Germany) and the primers 5′-ATC GGC TAG CCA GTT TCC AGG CTG CAC GTA-3′ and 5′-ATC GCT CGA GTC TTC TCA GTT GCT CGC TGC-3′. A 1.1-kb fragment containing *Nhe*I and *Xho*I sites was then ligated into the PCRII-TOPO vector by using a TA cloning kit (Invitrogen). The insertion fragment was sequenced by using SP6 and T7. The promoter fragment was excised from PCRII-TOPO-*c-fos* by digesting it with *Nhe*I and *Xho*I, and then cloned to the pGL3-basic vector to produce the pGL3-*c-fos* promoter. The predicted κB site at −216/−206 of the mouse *c-fos* promoter region (GGAGACCCCC) was mutated to GGAGAACACG by using the QuikChange Site-Directed Mutagenesis Kit from Agilent Technologies (Santa Clara, CA, USA). The NF-κB reporter plasmid containing 3 κB-binding sites (pGL2-ELMA-luciferase) was provided by Dr. S. L. Hsieh (Yang-Ming University, Taipei, Taiwan).

Following the commercial standard protocol, HEK293 cells and MEFs were cotransfected with a reporter plasmid and a β-galactosidase expression vector (pSVlacZ) by using Lipofectamine 2000 (Invitrogen). After treatment with the indicated reagents, the cells were lysed in a reporter lysis buffer (Promega) and the lysates were reacted with a commercial luciferase substrate provided in the luciferase assay system kit (Promega) and assayed on a microplate luminometer (Packard, Meriden, CT, USA). Luciferase activity was normalized relative to the activity of β-galactosidase and expressed as multiples of the untreated control. In some experiments, p65^−/−^ MEFs were transfected with HA-p65 by using Lipofectamine 2000.

### Chromatin Immunoprecipitation (ChIP) Assay

In vivo binding of the p65 transcription factor to the mouse *c-fos* promoter was investigated using a ChIP assay according to the EZ-CHIP™ protocol (Merck KGaA, Darmstadt, Germany). MEFs (3×10^6^) were treated for 10 min with 1% formaldehyde diluted in a tissue culture medium at 37°C and then washed twice in ice-cold PBS containing protease inhibitors (1 mM PMSF and 1 µg/mL each of aprotinin and pepstatin A). The cells were pelleted and lysed in an SDS lysis buffer. The samples were sonicated to shear the DNA to lengths of 200–1000 bp and were then diluted 5-fold with a dilution buffer (0.01% SDS, 1.2 mM EDTA, 16.7 mM Tris-HCl at pH 8.1, 1.1% Triton X-100, and 167 mM NaCl). The diluted cell lysates were precleared using salmon-sperm DNA/protein A agarose for 1 h at 4°C and then incubated with 2 µg of a specific antibody or normal immunoglobulin G (IgG) overnight at 4°C to immunoprecipitate protein-DNA complexes from the precleared supernatants; immune complexes were precipitated by using protein A agarose beads. The immunoprecipitated DNA was amplified by performing qPCR using an ABI Prism 7900. In the PCR, primers flanking the κB-binding sites in the mouse *c-fos* promoter were used, and a 200-bp *c-fos* promoter fragment was detected using sense (5′- GGG ACC ATC TCC GAA ATC CTA CAC GC-3′) and antisense (5′- GGA CTT CCT ACG TCA CTG GGC GGA AC-3′) primers corresponding to nucleotide positions −250 → −50 of the mouse *c-fos* promoter. Furthermore, we conducted a positive ChIP assay to confirm the stimulating effects of TNF-α on the κB site. The following primers around the κB-binding site in the ICAM-1 promoter were designed: sense 5′-GAT GTC CTT TCC GGT TGC AG-3′ and antisense 5′-CAG GGA AAT TCC CGG AGT ACA-3′ [26]. To verify the experimental specificity of the ChIP assay, we chose as a negative control a DNA site lacking the NF-κB-binding element that is present in the Igκ promoter [27]; the negative control primers in the Igκ gene promoter were the following: sense 5′-CTA ATA GTC CCA TGC TCT CC-3′ and antisense 5′-TCC TTC CTT TGG TGG CTT C-3′.

### Small Interfering (si)RNA

HEK293 cells (3×10^6^) were transfected with 100 nM siRNA targeting the mRNA of human p65 (catalog no. L-003533-00) by using DharmaFECT Transfection Reagents. The control nontargeting siRNA used was a pool of 4 functional nontargeting siRNAs. All siRNAs were obtained from Dharmacon Research (Lafayette, CO, USA). After siRNA transfection for 48 h, the cells were treated with various stimulants and the gene-silencing effects were then evaluated by performing immunoblotting assays.

### Statistical Evaluation

Values are expressed as the mean ± the standard error of the mean (SEM) of at least 3 independent experiments, which were performed in duplicate. An analysis of variance (ANOVA) was used to assess the statistical significance of differences, and *P*<0.05 was considered statistically significant.

## Results

### PMA, but not TNF-α, Induced c-Fos Expression and Elk-1 Phosphorylation in MEFs and HEK293 Cells

**Figure 1:**
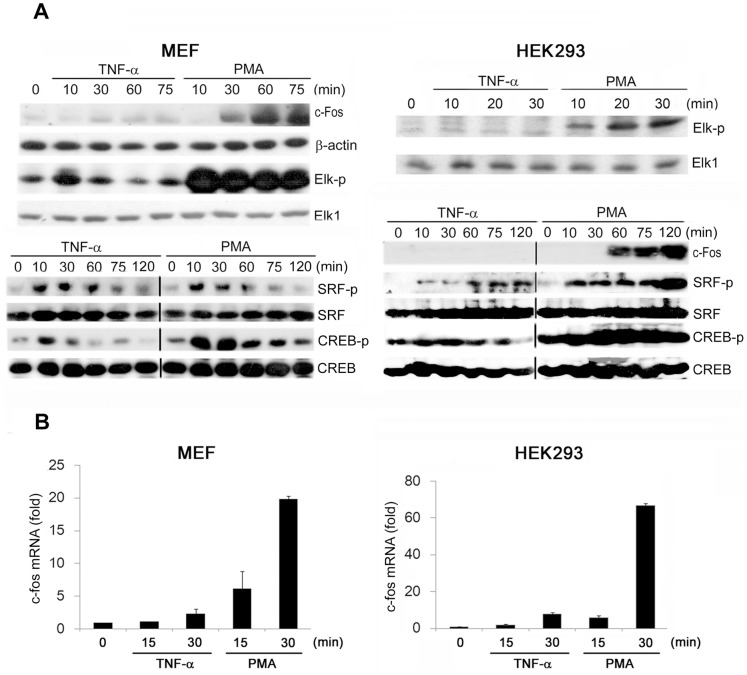
PMA but not TNF-α induces c-Fos expression in MEFs and HEK293 cells. MEFs and HEK293 cells were treated with TNF-α (20 ng/mL) or PMA (10 nM) for the indicated times. (A) Total cell lysates were prepared and subjected to western blotting analysis; the antibodies used are indicated. The western blots are representative of 3 independent experiments. (B) Real-time PCR assays were performed to detect *c-fos* mRNA expression. Data are the means ± SEM from 3 independent experiments.

PMA, which is structurally similar to diacylglycerol, is a potent PKC activator that is widely used to study the cellular functions of PKC. In MEFs and HEK293 cells, we first compared the effects of PMA and the proinflammatory cytokine TNF-α on c-Fos expression. We determined that PMA (10 nM) robustly upregulated c-Fos protein and gene expression in MEFs and HEK293 cells in a time-dependent manner; by contrast, TNF-α (20 ng/mL) did not similarly upregulate c-Fos expression (Fig. 1A, B). Because c-Fos induction requires the coordinated binding of TCF and SRF to SRE and CREB binding to CRE, we subsequently tested whether stimulating cells with the 2 reagents alters the phosphorylation of Elk-1 (a major TCF member), SRF, and CREB. Both PMA and TNF-α induced Elk-1 phosphorylation in MEFs and HEK293 cells, but the extent of stimulation, which was correlated with their effectiveness in inducing c-Fos, was considerably stronger and sustained with PMA treatment than with TNF-α treatment (Fig. 1A). PMA also elicited stronger and longer-lasting CREB phosphorylation than TNF-α did in MEFs and HEK293 cells. In HEK293 cells, TNF-α did not induce significant CREB phosphorylation (Fig. 1A). By contrast, PMA and TNF-α induced SRF phosphorylation to a similar extent and with similar kinetics in MEFs, and PMA induced slightly higher SRF phosphorylation than TNF-α did in HEK293 cells (Fig. 1A). These results suggest that TNF-α is a poorer inducer of c-Fos than PMA in MEFs and HEK293 cells, and that Elk-1 and CREB might act as major determinants of c-Fos induction.

### NF-κB is Involved in PMA-induced c-Fos Expression

**Figure 2:**
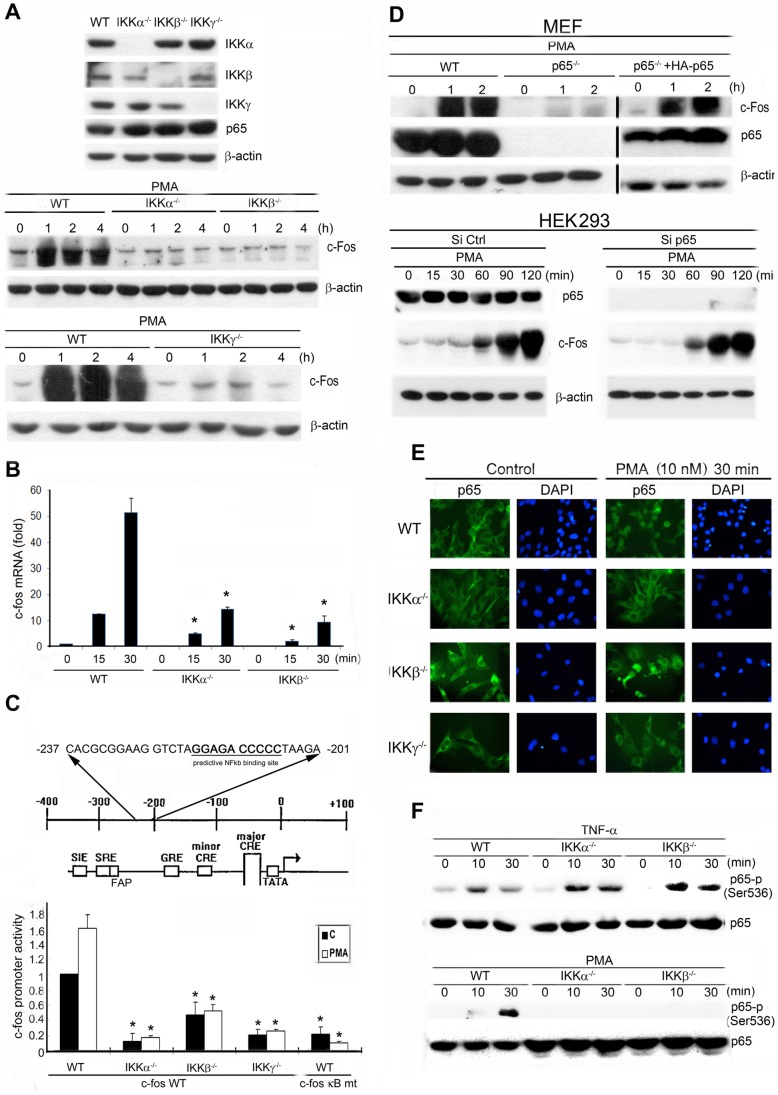
The IKK complex is involved in PMA-induced p65 phosphorylation and nuclear localization and c-Fos expression. In most experiments, WT, IKKα^−/−^, IKKβ^−/−^, IKKγ^−/−^, and p65^−/−^ MEFs were treated with TNF-α (20 ng/mL) or PMA (10 nM) for the indicated times. (A, D, F) Total cell lysates were prepared and subjected to western blotting analysis; the antibodies used are indicated. The western blots are representative of 3 independent experiments. (B) Total RNA was extracted, reverse transcribed into cDNAs, and subjected to qPCR analysis. Values were normalized relative to β-actin gene expression and expressed relative to the control sample. * *P*<0.05 indicates a significant reduction in PMA-induced gene expression in IKK-null MEFs. (C) Distinct response elements and the predicted NF-κB-binding site in the mouse *c-fos* promoter are shown. Regulatory elements identified in the mouse *c-fos* promoter are labeled and include the cis-inducible elements SRE, Fos activator protein 1 site (FAP), a glucocorticoid response element (GRE), and CRE. The major CRE is nearest of the sites to the start site within the large box, whereas the minor CREs are further upstream and are denoted using small boxes. Constitutive activity of the *c-fos* promoter with or without mutation of the κB site was determined in WT and IKK-null MEFs. Data in (B) and (C) are the means ± SEM from 3 independent experiments. * *P*<0.05 indicates a significant reduction in basal and PMA-induced promoter activity in IKK-null MEFs. (D) The p65^−/−^ MEFs were reconstituted with p65 protein, and HEK293 cells were transiently transfected with control or p65 siRNA (100 nM) for 48 h. Cells were treated with PMA for the indicated times. (E) Immunofluorescent staining of p65 (green) and nuclei (blue) shown by images captured under a fluorescence microscope; similar results were obtained in 2 additional experiments.

We subsequently investigated the link between NF-κB and c-Fos by comparing the effects of PMA on WT, IKKα^−/−^, IKKβ^−/−^, and IKKγ^−/−^ MEFs. We confirmed the deficiency of each IKK isoform (α, β, and γ) in the respective IKK-null MEFs and established that p65 was expressed at equal levels in these cells. Our results showed that the marked upregulation of c-Fos protein induced by PMA was abrogated in IKKα^−/−^, IKKβ^−/−^, and IKKγ^−/−^ MEFs (Fig. 2A). We used real-time PCR to determine whether this inhibition occurred through a transcriptional or a translational mechanism. We observed that *c-fos* mRNA levels increased substantially within 15–30 min after treatment of WT MEFs with PMA, but this effect was severely diminished in IKKα^−/−^ and IKKβ^−/−^ MEFs (Fig. 2B). Supporting this finding, *c-fos* promoter activity was reduced in all 3 types of IKK-null MEFs, which suggests that constitutive NF-κB activity is required for *c-fos* expression. To examine this possibility more closely, we analyzed the mouse *c-fos* promoter by using the Transcription Element Search System (TESS) and Signal Scan and identified an NF-κB binding site (with a TESS La score of 10.7494) that lies at −206 to −216 in the proximal region of the mouse *c-fos* promoter (Fig. 2C, upper panel). Mutation of the κB -binding sequence reduced the basal promoter activity to 30% of the WT promoter level, and PMA no longer elicited *c-fos* promoter activation (Fig. 2C, lower panel). Based on these results, we concluded that the κB-binding site is a critical cis-element that controls PMA-induced mouse *c-fos* promoter activity.

To further confirm the role of NF-κB in *c-fos* expression, we tested the PMA-induced c-Fos response in p65^−/−^ MEFs and p65-silenced HEK293 cells. We determined that PMA-induced c-Fos protein expression was suppressed in p65^−/−^ MEFs. Conversely, the loss of c-Fos expression was restored when p65 was ectopically expressed in p65^−/−^ MEFs (Fig. 2D, upper panel). However, silencing p65 in HEK293 cells did not affect PMA-induced human c-Fos protein expression (Fig. 2D, lower panel). These results indicate that p65 plays a critical role in the PMA-induced expression of mouse but not human c-Fos.

To examine p65 localization, we used immunofluorescence microscopy. In both WT and IKK-null MEFs, p65 was localized in the cytoplasm before PMA stimulation. After PMA treatment for 30 min, WT MEFs clearly displayed nuclear localization of p65, whereas IKKα^−/−^ and IKKγ^−/−^ MEFs did not exhibit p65 localization in nuclei. In IKKβ^−/−^ MEFs, although PMA-induced translocation of p65 was markedly reduced, we still detected nuclear p65 in approximately 30% of the population, compared with >90% in the case of WT cells (Fig. 2E). These results suggest that PMA-induced p65 nuclear translocation required each member of the IKK complex, especially IKKα and IKKγ.

In addition to the nuclear translocation of p65 following IκBα downregulation, direct IKK-targeted p65 phosphorylation at Ser536 was shown to be critical for p65 nuclear translocation and, in turn, for NF-κB transactivation [28], [29]. To test whether IKK isoforms exhibit this activity in response to PMA and TNF-α, we examined their effects on Ser536 phosphorylation in p65. TNF-α induced p65 phosphorylation on Ser536, and this was not altered in IKKα^−/−^ or IKKβ^−/−^ MEFs (Fig. 2F, upper panel). PMA also induced p65 (Ser536) phosphorylation, but unlike in the case of TNF-α, this phosphorylation was markedly reduced in MEFs deficient in each of the IKK isoforms (Fig. 2F, lower panel). Thus, we suggest that, whereas TNF-α-induced p65 (Ser536) phosphorylation occurs through a pathway independent of IKKα/β, the IKKs play key roles in PMA-induced p65 (Ser536) phosphorylation.

### PMA Induces Less IKK Activation than does TNF-α

**Figure 3:**
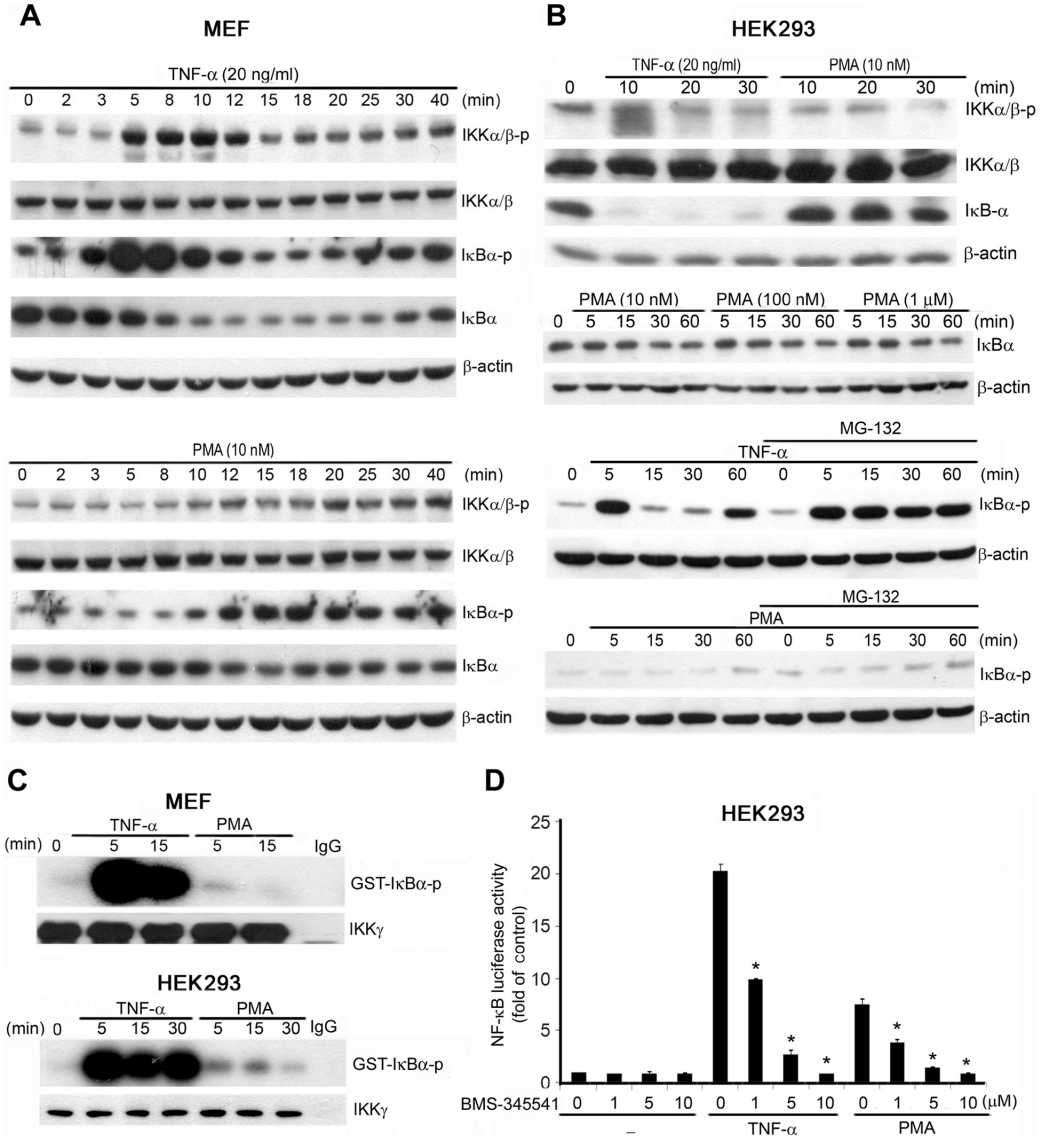
PMA induces weaker IKK and NF-κB activation than does TNF-α. MEFs (A) and HEK293 cells (B) were treated with TNF-α (20 ng/mL) or PMA (10 nM) for the indicated periods. In some experiments (B), HEK293 cells were pretreated with MG-132 (10 µM) for 30 min prior to treatment with PMA at the concentration indicated or with TNF-α (20 ng/mL) for various periods. Total cell lysates were collected and subjected to western blotting analysis; the antibodies used are indicated. The western blots are representative of 3 independent experiments. (C) After treatment with TNF-α (20 ng/mL) or PMA (10 nM) for the indicated times, IKK complex activity was determined using an in vitro kinase assay in which GST-IκB was included as the substrate. The autoradiograph shown here is representative of 3 independent experiments. (D) HEK293 cells transfected with the κB reporter andβ-galactosidase plasmids were treated with the IKK inhibitor BMS-345541 (1–10 µM) for 30 min and then stimulated with TNF-α (20 ng/mL) or PMA (10 nM) for 8 h. NF-κB luciferase activity was determined and normalized relative toβ-galactosidase activity. Data are the means ± SEM from 3 independent experiments. * *P*<0.05 indicates significant inhibition of TNF-α and PMA responses after treatment with BMS-345541.

After determining that the IKK complex and p65 were required for PMA-induced c-Fos expression, we determined the effect of PMA on IKK-dependent NF-κB activation and compared that with the effect of TNF-α. In MEFs, TNF-α treatment increased IKKα/β (Ser176/177) and IκBα phosphorylation within 5 min and sustained IKKα/β (Ser176/177) phosphorylation for 40 min. Concurrently, IκBα downregulation, an index of IKK-mediated IκBα phosphorylation, occurred after 5 min, but recovered by 40 min. Compared with TNF-α, PMA (10 nM) induced the phosphorylation of IKKα/β (Ser176/177) and IκBα more slowly and to a lesser extent (Fig. 3A). Similar disparities in PMA- and TNF-α-dependent IKK activation were also detected in HEK293 cells. TNF-α increased IKKα/β phosphorylation at Ser176/177 by 10 min, and this was accompanied by IκBα degradation. By contrast, PMA (10 nM) induced neither IKKα/β (Ser176/177) phosphorylation nor IκBα degradation (Fig. 3B), and increasing PMA concentrations up to 1 µM did not result in an increase in IκBα degradation (Fig. 3B). Because IκBα degradation is mediated by the proteasome degradation system, we pretreated cells with the proteasome inhibitor MG-132 (10 µM) and then examined the effects of the 2 stimuli. Treatment with MG-132 sustained IκBα phosphorylation in response to TNF-α, but did not cause a clear elevation of IκBα phosphorylation after PMA treatment (Fig. 3B).

To confirm that the limited effect of PMA on IκBα could be ascribed to weak activation of IKK, we conducted an in vitro kinase assay by using the IKK complex isolated from HEK293 cells and MEFs. TNF-α rapidly induced GST-IκBα (1–67) phosphorylation, and this occurred in parallel with a TNF-α-dependent increase in IKK phosphorylation and IκBα degradation. Compared with the clear phosphorylation of GST-IκBα(1–67) in response to TNF-α stimulation, the IKK complex showed poor kinase activity toward its substrate after treatment with PMA (Fig. 3C). Collectively, these results suggest that PMA weakly stimulates the IKK activity required for IκBα phosphorylation and downregulation. In agreement with this notion, when we determined NF-κB activity by measuring the κB reporter activity, we observed that TNF-α induced higher NF-κB luciferase activity in HEK293 cells than did PMA, and both actions were blocked by the IKK inhibitor BMS-345541 in a concentration-dependent manner (Fig. 3D). These results strongly indicate that, compared with the potent activator TNF-α, PMA produced a substantially weaker effect on the IKK-NF-κB signaling pathway in both MEFs and HEK293 cells.

### The p65 Homodimer Binds to a κB Site in the Mouse *c-fos* Promoter

**Figure 4:**
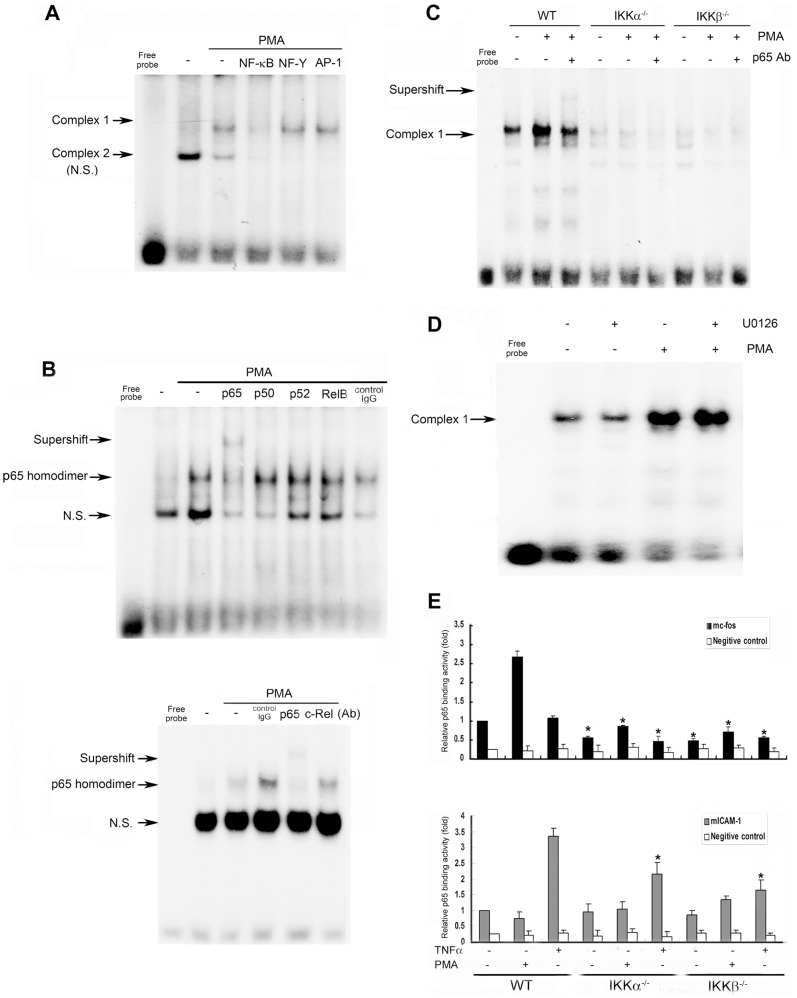
PMA stimulates p65 homodimer binding to the *c-fos* promoter. WT (A–D) and IKKα^−/−^ and IKKβ^−/−^ (C) MEFs were treated with PMA (10 nM) for 30 min, and nuclear extracts were prepared and assayed for binding activity by using specific oligonucleotides containing the predicted NF-κB binding site of the mouse *c-fos* promoter. (A) Consensus binding sequences of NF-κB, NF-Y, or AP-1 were used to confirm the specific NF-κB binding of Complex 1 and the nonspecificity of Complex 2. (B–C) Various antibodies were preincubated with nuclear extracts for 30 min to analyze binding specificity. (D) Cells were pretreated with U0126 (3 µM) for 30 min before adding PMA. Data in (A)–(D) are representative of 3 independent experiments. (E) WT and IKK-null MEFs were treated with PMA or TNF-α for 30 min and ChIP assays were then performed as described in the methods section. Data in (E) are the means ± SEM from 3 independent experiments. * *P*<0.05 indicates significant inhibition of p65 binding relative to the corresponding control responses in WT cells.

To elucidate the transcriptional function of NF-κB in regulating mouse *c-fos* expression, we designed oligonucleotides containing this NF-κB-binding site (−226 to −196) and conducted EMSAs. Two complexes were formed before and after PMA stimulation in WT MEFs (Fig. 4A). Complex 2 exhibited nonspecific binding because the binding was inhibited when we used cold probes of consensus NF-κB, NF-Y, and AP-1 sequences (Fig. 4A). By contrast, the Complex 1 signal that was enhanced after PMA stimulation was specific to NF-κB (Fig. 4A), and when the p65 antibody was added, a clear supershifted band beyond Complex 1 was detected (Fig. 4B), suggesting that p65 was bound to this complex. We also added antibodies against other NF-κB family members to identify the binding partner of p65. However, antibodies against p50, p52, RelB, and c-Rel did not produce a supershift after PMA stimulation (Fig. 4B), suggesting that the p65 homodimer is involved in *c-fos* regulation. Furthermore, nuclear extracts of PMA-treated IKKα^−/−^ and IKKβ^−/−^ MEFs failed to interact with oligonucleotides containing the NF-κB binding site (Fig. 4C), and the p65 homodimer that bound to the NF-κB-binding site of the mouse *c-fos* promoter was not affected by U0126 treatment (Fig. 4D). These results suggest that the binding of the p65 homodimer to the mouse *c-fos* promoter is required for PMA-dependent *c-fos* induction.

To verify that p65 binding is required for PMA-induced *c-fos* promoter activation, we then performed a ChIP assay by using a specific antibody against p65 to immunoprecipitate formaldehyde-fixed chromatin. Basal and PMA-induced increases in the binding of p65 to the κB site (−206 to −216) of the *c-fos* promoter were reduced in the absence of IKKα or IKKβ (Fig. 4E, upper panel), and TNF-α treatment was unable to induce p65 binding to the κB site of the *c-fos* promoter. To confirm that these distinct effects are gene specific, we used the κB site of the *ICAM-1* gene in the ChIP assay. TNF-α has been shown to be capable of upregulating *ICAM-1* expression in MEFs [30], [31]. As expected, TNF-α increased the binding of p65 to the *ICAM-1* promoter, and this effect was diminished in the absence of IKKs (Fig. 4E, lower panel). To demonstrate the specificity of the results of the ChIP assay, we used an Igκ gene sequence lacking the κB site as a negative control [27]. Neither PMA nor TNF-α affected the weak (approximately 20% of the specific binding) and non-specific effect of the p65 antibody on the Igκpromoter. Thus, we concluded that p65 can directly interact with the κB site of the mouse *c-fos* promoter in MEFs treated with PMA but not TNF-α.

### The ERK Signaling Pathway is a Prerequisite for c-Fos Expression

**Figure 5:**
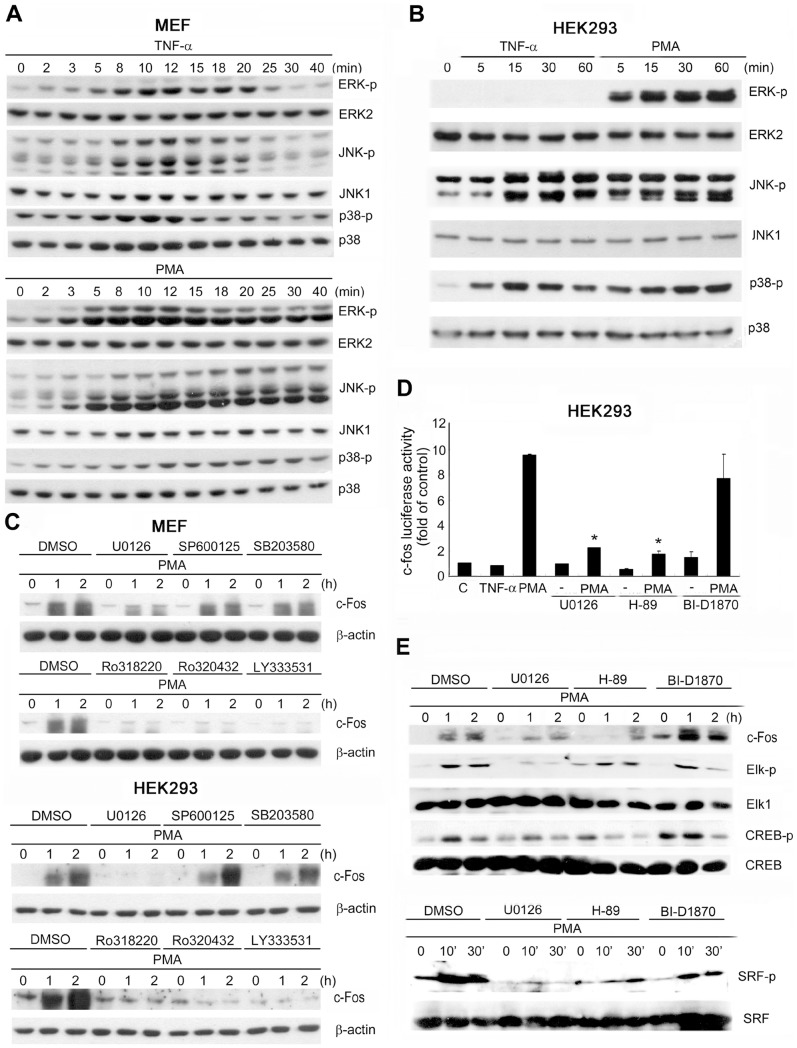
PKC-dependent MEK-ERK-MSK signaling is required for PMA-induced c-Fos expression. MEFs (A) and HEK293 cells (B) were treated with TNF-α (20 ng/mL) or PMA (10 nM) for the indicated times. (C) MEFs and HEK293 cells were pretreated with DMSO (0.1%), U0126 (3 µM), SP600125 (3 µM), SB203580 (3 µM), Ro318220 (3 µM), Ro320432 (1 µM), or LY333531 (1 µM) for 30 min, and then stimulated with PMA (10 nM) for the indicated times. (D) The activity of the *c-fos* promoter was determined in HEK293 cells treated with U0126 (3 µM), H-89 (10 µM), BI-D1870 (2 µM), PMA (10 nM), and/or TNF-α (20 ng/mL). (E) MEFs were pretreated with DMSO (0.1%), U0126 (3 µM), H-89 (10 µM), or BI-D1870 (2 µM) for 30 min, and then stimulated with PMA (10 nM) for the indicated times. Total cell lysates were prepared and subjected to western blotting analysis; the antibodies used are indicated. The western blots are representative of 3 independent experiments. Data in (D) are the means ± SEM from 3 independent experiments.

In addition to our observation that NF-κB contributes to *c-fos* expression, MAPKs have been reported to play key roles in controlling the induction of immediately early genes. Therefore, we examined the phosphorylation statuses of MAPKs in MEFs and HEK293 cells stimulated with TNF-α or PMA to determine whether transcription factors activated by MAPKs also contribute to the differential effects of TNF-α and PMA on *c-fos* induction. TNF-α and PMA induced the phosphorylation of ERK, p38, and JNK in MEFs (Fig. 5A) and HEK293 cells (Fig. 5B) to different extents. The major difference in the effects was that PMA was more potent than TNF-α in activating ERK, which was observed as a more sustained and dramatic increase in ERK phosphorylation in response to PMA than in response to TNF-α. By contrast, JNK and p38 were activated similarly by PMA and TNF-α.

To investigate the involvement of MAPKs and downstream signals in the induction of c-Fos expression, we used inhibitors of PKC and MAPKs. Treatment with PKC inhibitors (Ro318220, Ro320432, and LY333531) and a MEK inhibitor (U0126) abolished PMA induction of c-Fos protein expression in MEFs and HEK293 cells. However, p38 and JNK inhibitors (SB203580 and SP600125, respectively) did not alter the expression levels of c-Fos (Fig. 5C). Confirming these findings, *c-fos* promoter activity stimulated by PMA in HEK293 cells was blocked by the MEK inhibitor, U0126, whereas TNF-α did not increase *c-fos* promoter activity (Fig. 5D).

To test whether the signals downstream from ERKs were involved in PMA-induced c-Fos expression, MEFs were pretreated with inhibitors of MSK (H-89) and RSK (BI-D1870). Like U0126, only the MSK inhibitor clearly blocked PMA-induced *c-fos* promoter activation in HEK293 (Fig. 5D) and protein expression in MEFs (Fig. 5E). We also observed that Elk-1 phosphorylation was inhibited after treatment with U0126 but not H-89 or BI-D1870. By contrast, all inhibitors reduced SRF phosphorylation, and U0126 and H89 abolished this phosphorylation. Moreover, PMA-induced CREB phosphorylation was inhibited after the cells were treated with U0126 and H-89 (Fig. 5E). These results suggest that Elk-1 and CREB, but not SRF, play a major role in PMA-induced c-Fos expression.

### Independent Signaling Pathways of PMA-induced ERK and NF-κB Activation

**Figure 6:**
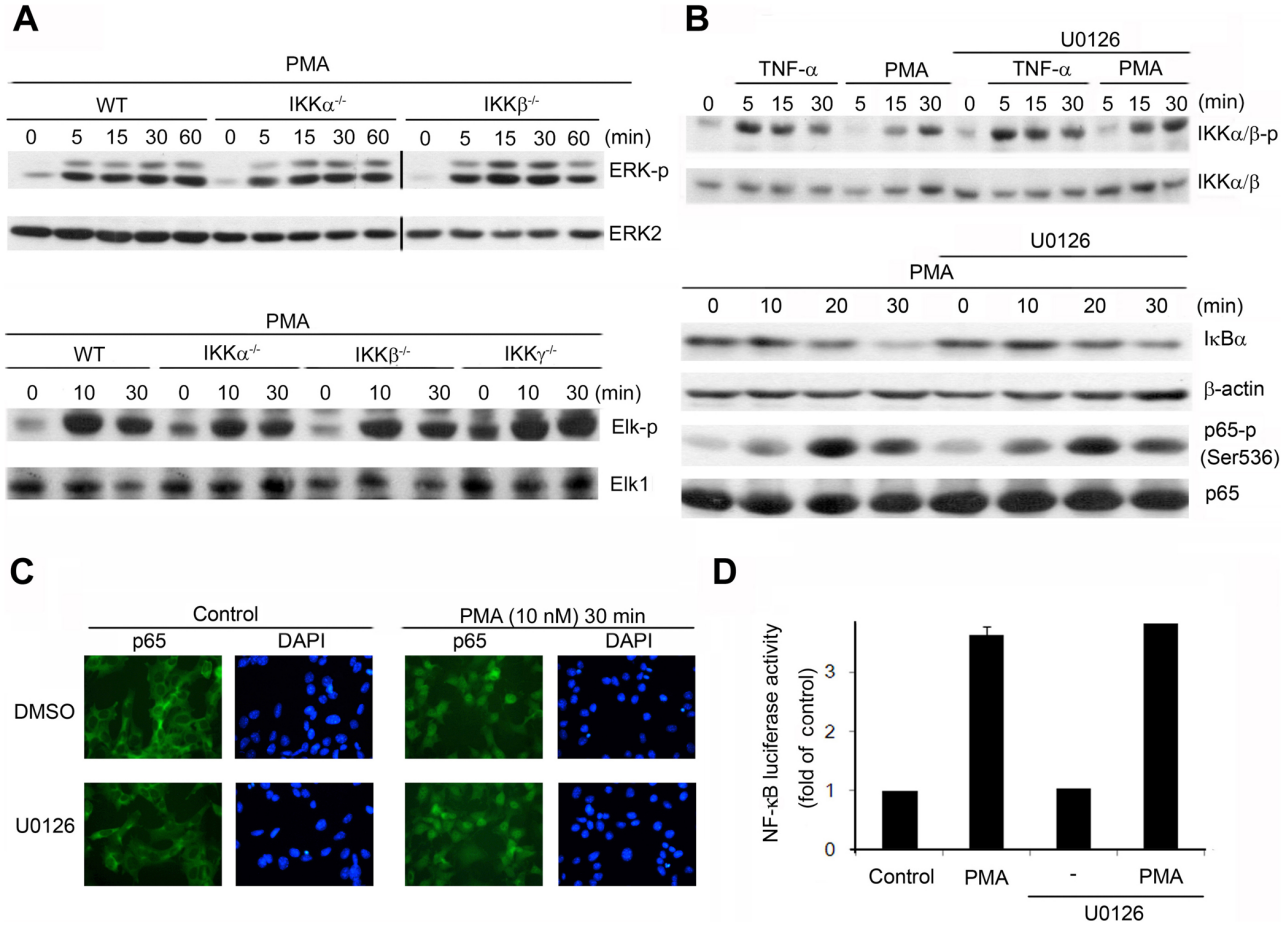
PMA-induced activation of ERK/Elk-1 and IKK/p65/NF-κB occurs through independent pathways. (A) WT, IKKα^−/−^, IKKβ^−/−^, and IKKγ^−/−^ MEFs were stimulated with PMA for various periods. (B) WT MEFs were pretreated with either DMSO (0.1%) or U0126 (3 µM) for 30 min and then stimulated with PMA (10 nM) for the indicated periods. Total cell lysates were prepared and subjected to western blotting analysis; the antibodies used are indicated. (C) The localization of p65 (green) and nuclear staining (blue) were determined by using fluorescence microscopy. (D) HEK293 cells transfected with the NF-κB luciferase reporter and theβ-galactosidase plasmid were pretreated with DMSO (0.1%) or U0126 (3 µM) for 30 min and then stimulated with PMA (10 nM) for 6 h. The luciferase activity derived from NF-κB activation was normalized relative to the transfection efficiency based on β-galactosidase. Data in (A)–(C) are representative of 3 independent experiments. Data in (D) are the means ± SEM from 3 independent experiments.

After determining the involvement of ERK-Elk and IKK in PMA-induced c-Fos expression, we assessed the potential crosstalk between IKK and ERK signaling in mediating c-Fos expression. The phosphorylation status of ERK was examined in IKK-null MEFs stimulated with PMA. ERK activation in response to PMA in IKKα^−/−^ and IKKβ^−/−^ MEFs was comparable to that in WT MEFs (Fig. 6A), and no defect in PMA-induced Elk-1 phosphorylation was observed in IKKα^−/−^, IKKβ^−/−^, or IKKγ^−/−^ MEFs (Fig. 6A). These results suggest that PMA activated ERK and Elk-1 independently of IKKs. Conversely, we also noted that U0126 did not affect TNF-α- or PMA-induced IKKα/β (Ser176/177) phosphorylation when ERK activity stimulated by PMA was inhibited (Fig. 6B). Neither PMA-induced IκB degradation nor p65 phosphorylation was altered in the presence of U0126 (Fig. 6B), and pretreatment with U0126 also did not alter PMA-induced p65 nuclear localization in MEFs (Fig. 6C). Furthermore, U0126 treatment did not affect PMA-induced NF-κB luciferase activity in HEK293 cells (Fig. 6D).

## Discussion

### PMA-induced IKK Activation and IκBα Phosphorylation were Weaker than those Induced by TNF-α

IKKα/β phosphorylation at Ser176/177 has been demonstrated previously to contribute to enzyme activation and induction of NF**-**κB signaling. In this study, we compared the abilities of TNF-α and PMA to activate IKK and NF-κB. TNF-α was determined to be a stronger activator of IKKα/β-NF-κB than PMA based on the following data. First, in both MEFs and HEK293 cells, TNF-α induced more efficient IκBα phosphorylation at Ser32 than PMA did. Ser32 is a well-known IKKβ target that constitutes the major element of canonical NF-κB activation. Second, proteasome-mediated degradation of IκBα that accompanied the protein’s phosphorylation was more clearly detected in cells treated with TNF-α than in cells exposed to PMA. Third, consistent with the effect on IκBα, measurement of in vitro IκBα phosphorylation demonstrated that TNF-αinduced robust IKK activity in HEK293 cells and MEFs. By contrast, the effects of PMA were minimal. Fourth, stronger IKK-dependent NF-κB activity was detected in a reporter assay in response to TNF-α than in response to PMA.

### IKKα and IKKβ Mediate PMA-induced p65 Phosphorylation, Nuclear Translocation, and NF-κB Activation

In addition to the nuclear translocation of p65 that is required for NF-κB activation, the phosphorylation of p65 at Ser536 participates in NF-κB activation. The phosphorylation of p65 (Ser536), which was proposed to be mediated by numerous kinases, including IKKα/β, p38, RSK1, TBK1, and Akt [32]–[37], occurs within the transactivation domain of p65 and may induce a conformational change that lower the interaction of p65 with IκBα, the protein that mediates NF-κB nuclear exportation [33], [34]. Moreover, the phosphorylation of p65 at Ser536 also results in the displacement of the corepressor SMRT-HDAC3 complex and increases p300/CBP recruitment [38]. These findings collectively indicate the existence of an alternative NF-κB activation pathway that is independent of IKKβ-mediated IκB degradation.

In this study, we observed that both PMA and TNF-α induced p65 (Ser536) phosphorylation within 30 min. TNF-α-induced p65 (Ser536) phosphorylation showed no defects in IKKα^−/−^ and IKKβ^−/−^ MEFs, but was inhibited by treatment with SB203580 (data not shown), suggesting that p38 contributes to this phosphorylation. By contrast, a deficiency in each of the IKK isoforms potently reduced PMA-induced p65 phosphorylation and, accordingly, PMA-induced p65 nuclear localization was impaired in IKKα^−/−^ and IKKγ^−/−^ MEFs. Because PMA-induced IκBα phosphorylation was not clearly detected in MEFs, we suggest that the nuclear localization of p65 induced by PMA, unlike that caused by treatment with TNF-α, does not result from IKK-mediated IκB degradation, but can be ascribed to IKKα/β-mediated p65 phosphorylation.

### NF-κB Signaling Coordinates with ERK-Elk-1 and ERK-MSK-CREB Signaling to Mediate *c-fos* Expression

The TCF-SRF complex has been demonstrated to play a critical role in *c-fos* expression [39]. Similarly, MSK-dependent CREB activation has been shown to be involved in *c-fos* expression [2], [15]. Elk-1 is a component of TCF and the DNA-binding capacity of Elk-1 increases after being phosphorylated on Ser383 in its C domain, thus undergoing conformational changes [40]. Phosphorylation of Elk-1 regulates its DNA binding and coordination with SRF for the transcriptional activation of *c-fos* [1], [8], [10]. In this study, we determined that Elk-1 and CREB activated by ERK and the ERK downstream target MSK, respectively, were essential for *c-fos* expression. The considerably weaker effect of TNF-α than of PMA on both Elk-1 and CREB activation accounts for the minimal ability of TNF-α to induce c-Fos. Furthermore, unlike the serum-induced c-Fos expression that requires SRF and MKL1, a potent transcriptional coactivator of SRF [14], [20], the action of PMA action might be independent of SRF (see below).

How NF-κB contributes to *c-fos* expression remains poorly understood. EGF was shown to induce c-Fos expression in MEFs through an IKK-dependent mechanism [20]. Another study showed that Elk-1 is an NF-κB-regulated gene [19]. In this study, we demonstrated a crucial role of NF-κB in mouse c-Fos upregulation. First, we noted that PMA-induced c-Fos expression at both the gene and protein levels was markedly diminished in IKK- and p65-null MEFs, which suggests that NF-κB regulates c-Fos expression. Second, our data revealed that the ERK-Elk-1 and IKK-p65-NF-κB axes independently regulated c-Fos expression. Neither ERK nor Elk-1 activation in response to PMA was affected in IKK-null MEFs. Moreover, applying U0126 did not affect the PMA induction of events that depend on NF-κB activation: IKK activation, IκBα degradation, and p65 phosphorylation, nuclear translocation, and DNA binding and activation. Third, we used TESS to identify a previously uncharacterized NF-κB-binding site in the −206 to −216 region of the mouse *c-fos* promoter. Analyzing DNA binding by using EMSA and ChIP assays demonstrated that the p65 homodimer can bind to this promoter region. A specific DNA-recognition mode of the NF-κB p65 homodimer that is distinct from the classical binding mode of the p65-p50 heterodimer has been identified structurally [41], [42]. Functionally, the p65 homodimer was shown to be present in PMA-stimulated Jurkat T cells [43] and was identified to bind the promoter of *IL-8* [44]. In addition to this effect of PMA, the critical roles of the p65 homodimer in biological function have been demonstrated, such as in inducing neutrophilic inflammation in airway cells [45] and in mediating ICAM-1 expression and neutrophil adhesion in endothelial cells [46]. Thus, the binding of the p65 homodimer to the mouse *c-fos* promoter is another example that supports the function of the p65 homodimer in transcriptional control of genes. A key finding in this study was that the NF-κB-binding site identified in the mouse *c-fos* promoter is not present in the human gene. However, Fujita et al. demonstrated that the NF-κB p50-binding site in the first intron of *c-fos* contributes to its expression in response to TNF-α [47]. Currently, we cannot exclude this possibility and thus must further investigate whether or not the intragenic NF-κB binding site is involved in PMA-induced c-*fos* expression in future studies. Moreover, in contrast to a report showing that ERK-dependent recruitment of NFI to the human *c-fos* promoter is required for gene expression [48], we observed no NFI-binding site in the mouse *c-fos* promoter. Thus, ERK-NFI and NF-κB regulate *c-fos* expression differently in mice and humans.

This study also showed that NF-κB activation alone is insufficient for inducing c-Fos and that this induction requires adequate ERK-mediated Elk-1 and CREB phosphorylation. This conclusion is primarily based on the action of TNF-α. Although TNF-α was a considerably stronger activator of NF-κB than PMA in MEFs and HEK293 cells, TNF-α poorly induced c-Fos, which can be explained by the inability of TNF-α to induce strong and sustained activation of ERK and the essential downstream transcription factors Elk-1 and CREB. Moreover, because TNF-α could not induce NF-κB binding to the *c-fos* promoter, we suggest that an additional regulator is required for PMA to stimulate the transcriptional function of NF-κB, and that NF-κB coordinates the activation of Elk-1 and CREB in the upregulation of mouse *c-fos* transcription.

We demonstrated that ERK was required for Elk-1 phosphorylation and activation, but that the downstream kinases MSK and RSK were not required. In addition to Elk-1 phosphorylation, which plays an indispensable role in c-Fos protein expression (as mentioned above), the contribution of SRF phosphorylation to c-Fos expression remains disputed. In PMA-stimulated MEFs, we determined that SRF phosphorylation was suppressed by inhibitors of ERK (U0126), MSK (H-89), and RSK (BI-D1870), although only the ERK and MSK inhibitors attenuated c-Fos expression. Thus, we suggest that the phosphorylation of SRF by multiple kinases is not required for PMA-induced c-Fos expression. Moreover, because TNF-α induced SRF phosphorylation for a longer period than it activated ERK, we suggest that an ERK-independent kinase is involved in the TNF-α-dependent phosphorylation of SRF. Because no specific inhibitor of MSK is currently available and H-89 inhibits kinases other than MSK [49], [50], the H-89 data must be interpreted with caution.

**Figure 7:**
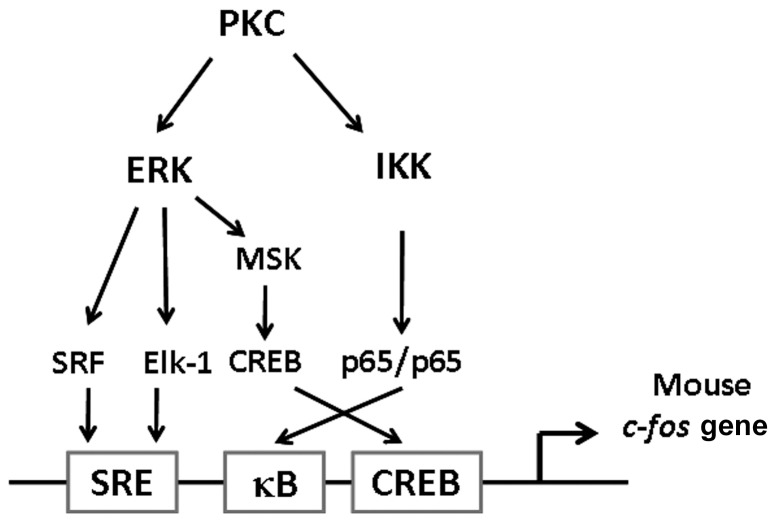
Schematic summary of the transcriptional regulation of mouse c-Fos gene expression. In addition to the recognized ERK-stimulated pathways required for c-Fos expression, the binding of the p65 homodimer to the κB site and the functioning of p65 in a coordinated manner with the transcription factors Elk-1, SRF, and CREB is prerequisite for mouse PMA-induced c-Fos gene expression.

In summary, this study provides strong evidence that links NF-κB activation to c-Fos induction. We have demonstrated for the first time that, in addition to the widely recognized requirement of the ERK-Elk-1 and ERK-MSK-CREB signaling pathways in the control of c-Fos expression, the p65 homodimer, which bound to the *c-fos* promoter when stimulated by PMA, is indispensable for mouse *c-fos* transcription. This mechanism relies mainly on the members of the IKKα/β/γ complex and on their enzymatic-activity-mediated p65 phosphorylation and nuclear localization (Fig. 7). However, the NF-κB activation that occurs without sufficient Elk-1 and/or CREB phosphorylation under TNF-α stimulation does not upregulate c-Fos. These results provide key evidence linking 2 vital transcription systems (NF-κB and c-Fos) in the control of various aspects of cellular functions, and these findings warrant further investigation.
